# Notes and News

**Published:** 1903-03-21

**Authors:** 


### NOTES AND NEWS.

His Royal Highness the Duke of Cambridge, K.G., President of the Sanitary Institute, will preside at the annual dinner of the Sanitary Institute to be held on Friday, May 15th, at the Hotel Cecil.

The "Craggs Research Prize" of £50, in connection with the London School of Tropical Medicine, will be awarded in October to a past or present student of the school who makes the most valuable contribution to tropical medicine.

A bottle of soda water, recovered from the wreck of the *Royal George*, was sold by auction for £25 recently. The relic is said to be well authenticated, and as the *Royal George* was sunk on August 29th, 1782, it is over 120 years old. Some two-thirds of the contents, originally about half a pint, remain, the cork being still retained by wire partially displaced. Chemical changes corresponding to those produced by ullage in wine presumably account for a thin internal encrustation or discoloration of salts. Soda water was apparently sold in London by a Schweppe in 1798. A soda water bottle dug up on the Crimean battlefield is still in the possession of this firm.

Dr. O'Connor, Medical Officer of Health for Melton District, gives the real Melton Mowbray pies a very good character. He says in a report: "The wholesale trade in Melton Mowbray pork pies is essentially in the hands of three firms, on each of whose premises I seduously sought for every source of insanitation which could generate the slightest suspicion. I inspected the carcases, the method of storage, the method of cooking, the preparation of the pastry, and the method of filling the pies with the sterilised broth, and am in a position to authoritatively state that the pork pies exported from your town are as safe and wholesome a food as any similar article of diet made with most scrupulous care in one's home could possibly be."

The Rt. Hon. Lord Ludlow has kindly accepted the post of President and Chairman of the General Committee of the Cancer Hospital (Free), Fulham Road, S.W.

We are informed that the 1903 edition of "Burdett's Hospitals and Charities" will shortly be issued by The Scientific Press. This is the fourteenth annual issue of this valuable work, which is the recognised standard authority on all points relating to charities, whether British, American, or colonial, and may be regarded as the "Hospital Whitaker."

The twenty-first congress of the Sanitary Institute will be held at Bradford on July 7th to 11th under the presidency of the Right Hon. the Earl of Stamford. Section one—Sanitary Science and Preventive Medicine—will be presided over by Professor Clifford Allbutt; Section two — Engineering and Architecture—will be presided over by Maurice Fitzmaurice, C.M.G.; Section three — Physics, Chemistry, and Biology—will be presided over by Professor C. Hunter Stewart; and eight technical conferences will also be held in connection with the Congress.

It appears that the recent generous gift of £40,000 entrusted to Lord Cromer and his successors in office by Sir Ernest Cassel, for the relief of ophthalmia and eye diseases, and for the training of qualified men for such work in Egypt, was the direct outcome of the Khedive expressing interest in the proposal for an ophthalmic research hospital which had been submitted by Mr. Kenneth Scott, ophthalmic surgeon, of London, in the hope that the funds required for starting it might be provided. The Sanitary Department of the Egyptian Government, who will expend the sum, will establish a tent in the form of a "travelling dispensary," for purposes of operation and treatment, to work solely in the provinces. They will appoint an additional English inspector for a temporary period to travel with it, accompanied by a native Egyptian doctor, a post-graduate local medical student, two male hospital attendants, and two servants. Beyond the initial cost of about £250, its maintenance, inclusive of salaries, is estimated at £900 per annum.

